# Associations Between Body Composition Measurements and Muscle Ultrasound Parameters Amongst Children and Adolescents with Overweight and Obesity

**DOI:** 10.3390/nu17162659

**Published:** 2025-08-17

**Authors:** Andrea Domínguez-Barbosa, Dana Reyes-Romo, Mariel Salvador-Quezada, Sandra Nayeli Becerra-Morales, Desiree Lopez-Gonzalez, Aurora Elizabeth Serralde-Zúñiga, Martha Guevara-Cruz, Isabel Medina-Vera

**Affiliations:** 1 Departamento de Metodología de la Investigación, Instituto Nacional de Pediatría, Ciudad de México 04530, Mexico; andrea.dominguez@gmail.com (A.D.-B.); danareyesro@gmail.com (D.R.-R.); dra.marielsalvador@gmail.com (M.S.-Q.); nayelibm.052@gmail.com (S.N.B.-M.); 2 Facultad de Medicina, Programa de Doctorado en Ciencias Médicas, Odontológicas y de la Salud, Universidad Nacional Autónoma de México, Ciudad de México 04510, Mexico; 3 Laboratorio de Envejecimiento Saludable, Instituto Nacional de Medicina Genómica, Centro de Investigación Sobre Envejecimiento, Ciudad de México 14330, Mexico; 4 Dirección General de Calidad y Educación en Salud, Secretaría de Salud, Ciudad de México 11410, Mexico; 5 Unidad de Investigación en Epidemiología Clínica, Hospital Infantil de México Federico Gómez, Ciudad de México 06720, Mexico; dradesireelopez@gmail.com; 6 Servicio de Nutriología Clínica, Instituto Nacional de Ciencias Médicas y Nutrición Salvador Zubirán, Ciudad de México 14080, Mexico; aurozabeth@yahoo.com.mx; 7 Departamento de Fisiología de la Nutrición, Instituto Nacional de Ciencias Médicas y Nutrición Salvador Zubirán, Ciudad de México 14080, Mexico

**Keywords:** ultrasound, POCUS, echo intensity, body composition, childhood overweight, obesity

## Abstract

**Background/Objective:** Pediatric obesity negatively impacts metabolic and musculoskeletal health, particularly muscle quality and function. Ultrasound-derived measures like muscle thickness and echo intensity, combined with body composition data, provide a more comprehensive assessment of muscle status in this population. The purpose of our study was to examine the relationship between anthropometric measurements, muscle strength, and bioelectrical impedance estimations with ultrasound-derived indicators such as subcutaneous fat and quadriceps femoris thickness, as well as muscle quality, through EI. **Methods:** This cross-sectional study included Hispanic children aged 6 to under 18 years with overweight or obesity (BMI ≥ 85th percentile per CDC standards). Participants were recruited consecutively from outpatient visits. All eligible children were invited for a standardized nutritional assessment, and those who consented were included. **Results:** The study included 294 children and adolescents (153 boys, 141 girls) with overweight or obesity, showing significant sex differences in anthropometric and body composition variables. Girls had higher intramuscular adipose tissue (IMAT) (*p* < 0.001), while boys had more lean and musculoskeletal mass. Body fat percentage was significantly correlated with muscle echo intensity (EI corrected: R^2^ = 0.264, *p* < 0.001; EI uncorrected: R^2^ = 0.242, *p* < 0.001) and with IMAT (R^2^ = 0.268, *p* < 0.001). These associations were stronger in girls. Linear models identified body fat and BMI percentile as key predictors of muscle quality indicators (*p* < 0.001). **Conclusions:** This study found that higher body fat in children and adolescents with overweight or obesity is linked to poorer muscle quality, and especially increased echo intensity and intramuscular fat. Ultrasound proves useful for early, non-invasive detection of musculoskeletal changes, emphasizing the need to assess both muscle size and quality.

## 1. Introduction

The global surge in pediatric obesity represents one of the most pressing public health concerns of the 21st century. The implications of excess adiposity extend beyond metabolic disturbances, increasingly implicating musculoskeletal health, particularly muscle quality and function [1,2,3]. Several techniques are available to evaluate body composition in children, each with its own advantages and limitations. Dual-energy X-ray absorptiometry (DXA) is widely regarded as a reference method due to its precision in quantifying fat mass, lean mass, and bone mineral content; however, its clinical use in pediatrics is often limited by cost, access, and radiation exposure. Bioelectrical impedance analysis (BIA), on the other hand, offers a safe, quick, and non-invasive alternative that estimates body composition based on the conductivity of electrical currents through tissues. BIA is particularly advantageous in pediatric populations due to its feasibility in outpatient settings and its applicability across a wide age range. Despite its limitations in hydration sensitivity and variability among devices, BIA remains a valuable tool for obesity monitoring, especially when complemented by other modalities such as ultrasonography, which adds structural and qualitative muscle assessment [1,4,5,6]. Recent advances in imaging technologies, particularly ultrasound, have facilitated non-invasive, radiation-free assessments of muscle morphology and quality through parameters such as muscle thickness (MT) and echo intensity (EI) [1,3,7]. These ultrasound-derived indicators are gaining recognition as valuable tools for investigating obesity-related musculoskeletal alterations and for evaluating early risk factors for sarcopenic obesity [2].

Muscle thickness, as assessed by B-mode ultrasound, correlates strongly with muscle cross-sectional area and volume, making it a reliable surrogate of muscle mass in both adult and pediatric populations [8,9,10]. Likewise, echo intensity—a grayscale measure of tissue composition—has emerged as a proxy for muscle quality, with higher EI values typically reflecting increased intramuscular fat and fibrotic infiltration [5,11,12,13]. This qualitative degeneration has been linked to diminished strength, impaired function, and poor metabolic outcomes [4,6,14]. Importantly, in children and adolescents with obesity, the interpretation of EI is confounded by factors such as subcutaneous adipose tissue (SAT) thickness, signal attenuation, muscle hydration, and operator-dependent variability, raising important methodological and clinical questions [15,16,17,18]. Several studies have established that pediatric obesity is associated with increased visceral and intramuscular fat depots, contributing to metabolic derangements and muscle dysfunction [19,20,21]. Studies using cross-sectional and longitudinal designs have consistently demonstrated that increased EI is associated with poorer muscle performance, reduced cardiorespiratory fitness, and higher cardiometabolic risk factors [19,22,23,24,25]. Ultrasound has also shown promise in detecting early structural muscle impairments in obese children, even before overt functional declines become evident [26,27,28,29]. The rectus femoris muscle was selected for ultrasonographic evaluation due to its accessibility, reproducibility, and relevance in pediatric populations. This site has been consistently used in previous studies to assess muscle quality and intramuscular fat deposition in children and adolescents with obesity [15,23,27]. Assessing the right thigh allows for standardized positioning and minimizes inter-limb variability, which is particularly important in cross-sectional studies involving large samples. Research in diverse pediatric cohorts—including those with metabolic syndrome, non-alcoholic fatty liver disease (NAFLD), and insulin resistance—has further corroborated the link between EI and systemic metabolic disturbances [30,31,32,33]. The predictive validity of EI is supported by intervention studies showing that weight loss and resistance training can lead to measurable reductions in EI and improvements in strength and insulin sensitivity [34,35,36]. Despite this, methodological heterogeneity persists. Factors such as image acquisition technique, probe frequency, region of interest selection, and tissue hydration status can all influence EI values. Therefore, interpretation should be made in the context of standardized imaging protocols [37,38,39].

While muscle thickness has a more intuitive relationship with muscle volume and physical development, its utility in pediatric obesity is nuanced. Increased MT in children with obesity does not necessarily equate to greater function or metabolic benefit, as the presence of fat infiltration can obscure the relationship between size and quality [40,41,42]. The combined interpretation of MT and EI offers a more comprehensive approach for assessing muscle status; however, its role in association with body composition measurements in this population is scarce. The purpose of our study was to examine the relationship between anthropometric measurements, muscle strength, and bioelectrical impedance estimations, and ultrasound-derived indicators such as subcutaneous fat and quadriceps femoris thickness, as well as muscle quality, through EI.

## 2. Materials and Methods

### 2.1. Study Design and Participants

This cross-sectional study was conducted at the Instituto Nacional de Pediatría in Mexico City. Eligible participants were Hispanic children, both male and female, aged 6 < 18 years, with overweight or obesity, defined as a body mass index for age and sex at or above the 85th percentile according to CDC standards. Participants were selected using a non-probabilistic, consecutive sampling approach. Recruitment took place between 11 July 2024 and 15 April 2025 among patients with scheduled outpatient visits to the institute. All eligible individuals what attended during this period were invited to participate in a standardized nutritional assessment, and those who consented were included in the study. Exclusion criteria included a diagnosis of metabolic disorders other than overweight and obesity, recent trauma or surgery involving the upper and lower limbs, short stature for age, or current medical or pharmacological treatment of any kind.

### 2.2. Anthropometric Measurements

All participants underwent a standardized anthropometric evaluation during a single outpatient visit. Twelve kinanthropometric variables, as defined by the International Society for the Advancement of Kinanthropometry (ISAK) [43], were measured on the right side of the body. All measurements were conducted by two ISAK-certified Level I kinanthropometrists. The assessed variables included body weight, height, neck circumference, wrist circumference, relaxed and flexed mid-upper arm circumference, waist circumference, hip circumference, mid-thigh circumference, and mid-calf circumference. Body weight was measured in fasting participants wearing minimal clothing using a calibrated digital scale (SECA, Hamburg, Germany) to the nearest 0.1 kg. Height was measured with a stadiometer to the nearest 0.1 cm. Circumferences were measured in a plane orthogonal to the long axis of each body segment using a non-elastic fiberglass measuring tape, following standardized anatomical landmarks, and recorded to the nearest 0.1 cm. Neck circumference was measured immediately superior to the thyroid cartilage and perpendicular to the long axis of the neck; wrist circumference was measured with the participant holding the anterior surface of the right wrist upward, placing the tape distal to the prominences of the ulnar and radial bones without applying pressure; relaxed mid-upper arm circumference was measured at the level of the marked mid-acromiale–radiale site; tight mid-upper arm circumference was measured as the maximum girth of the right upper arm with the arm raised anteriorly to the horizontal and the forearm flexed at 90° to the upper arm; waist circumference was measured midway between the lowest rib and the top of the iliac crest at the end of a normal expiration; hip circumference was measured at the level of the greatest posterior prominence of the buttocks, perpendicular to the trunk’s long axis; mid-thigh circumference was measured perpendicular to the long axis of the thigh at the marked mid-trochanterion–tibiale laterale site; and mid-calf circumference was measured at the level of the greatest circumference of the calf.

Body mass index (BMI) was calculated as weight in kilograms divided by height in meters squared (kg/m^2^), and BMI-for-age percentiles were determined using age- and sex-specific reference data from the Centers for Disease Control and Prevention (CDC) growth charts [44]. Skinfold thickness was measured at marked anatomical sites using a Harpenden skinfold caliper, recorded to the nearest 0.1 mm. The triceps skinfold was measured at the midline of the posterior upper arm at the mid-acromiale–radiale landmark, while the subscapular skinfold was measured 2 cm along a line running laterally and obliquely downward at a 45° angle from the subscapulare landmark [43]. Each site was measured in triplicate, and the mean value was used for analysis. The sum of skinfolds was calculated by adding the triceps and subscapular values. Muscle strength was assessed using a Jamar hydraulic hand dynamometer (Jamar model J00105, Lafayette Instrument Company, Indiana USA; capacity 90 kg; weight 727 g) for the upper limbs and a back and leg dynamometer for the lower limbs (Takei 5002 Analogue, Takei Company, Tokyo, Japan; capacity 0–300 kg; weight 3.7 kg). Each participant performed two maximal voluntary contractions in a standardized position, and the highest value was recorded.

### 2.3. Body Composition

Body composition was measured using multifrequency bioelectrical impedance analysis (BIA) with a tetrapolar InBody S10 device (Model JMW140, Biospace Co., Ltd., Seoul, Republic of Korea). Prior to measurement, participants’ age, body weight and height were entered into the device, as per the manufacturer’s instructions. All assessments were performed with participants in a fasted state and under standardized hydration conditions and in supine position.

### 2.4. Ultrasonographic Assessment

**Figure 1:**
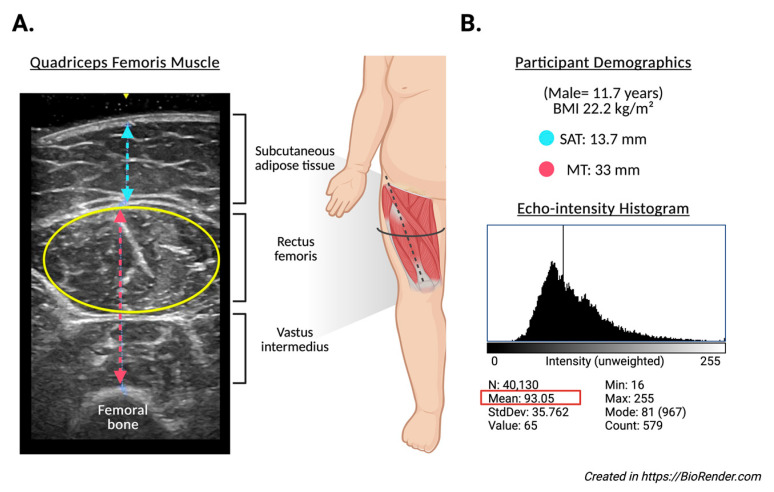
Representative ultrasound assessment of the quadriceps femoris muscle in an 11.7-year-old male participant with a BMI of 22.2 kg/m^2^. (**A**) displays the transverse B-mode ultrasound image, identifying subcutaneous adipose tissue (SAT), rectus femoris, and vastus intermedius muscles. The muscle thickness (MT) was measured at 33 mm (red line), and SAT thickness at 13.7 mm (blue line). The yellow ellipse indicates the region of interest (ROI) used for echo intensity analysis. (**B**) shows the corresponding echo intensity histogram derived from the ROI, with a mean grayscale value of 93.053 (SD: 35.762), and intensity values ranging from 16 to 255. These images exemplify the methodology for quantifying muscle morphology and quality in the study participants.

Ultrasound images of the assessment of the quadriceps femoris muscle (Figure 1A) were acquired using a portable B-mode imaging device (GE Healthcare, Ultrasound Venue System, Chicago, IL, USA) equipped with a 40 mm linear array probe, set to a frequency of 7.5 MHz, 80 mm scanning depth (adjusted in cases of greater subcutaneous fat), 70 dB gain, and maximum brightness and contrast. The scanning depth was only increased when testing participants with greater subcutaneous fat to allow for capturing enough muscle area. A high-frequency linear-array transducer was utilized for most of the assessments. In cases where full visualization of the muscle boundaries was not achievable due to excessive subcutaneous adiposity, a low-frequency convex transducer was employed to improve tissue penetration and ensure adequate delineation of muscle architecture. All acquisitions were performed with the participant in a supine position, with the right thigh exposed and the transducer placed transversely over the distal third of the thigh (midway between the anterior superior iliac spine and the superior margin of the patella). A clinical-grade water-based gel was applied to ensure adequate acoustic coupling. Triplicate static images were acquired from each subject under standardized conditions.

Echo intensity analysis (Figure 1B) was performed using ImageJ software (version 1.54g, National Institutes of Health, Bethesda, MD, USA). The largest visible cross-sectional area of the rectus femoris was manually defined using the oval selection tool. Within the defined region of interest (ROI), the echo intensity (EI) was computed as the mean gray-scale value from a histogram ranging from 0 (black) to 255 (white). To improve the reproducibility and efficiency of image processing, a custom macro script was developed and executed within the software platform. This macro automated the standardized application of a reference ROI to all images, extraction of grayscale histogram parameters (mean, standard deviation, area, minimum, maximum), and batch exportation of results into a structured .csv file. Additionally, the macro generated visual representations of histograms for each image, allowing consistent placement of ROIs and minimizing inter-operator variability. All images and measurements were independently reviewed by two trained evaluators. To control for the potential confounding effect of the subcutaneous fat on muscle EI, a corrected echo intensity variable was derived using a previously validated formula, adjusting EI values according to SAT thickness [45]. All measurements were performed by trained personnel using calibrated instruments and standardized protocols to ensure consistency and reliability.

### 2.5. Statistical Analysis

Continuous variables were tested for normality using the Kolmogorov–Smirnov test. Normally distributed variables were presented as mean ± standard deviation or mean with 95% confidence intervals, while non-normally distributed variables were reported as median and interquartile range (IQR), as appropriate. Categorical variables were expressed as frequencies and percentages. Anthropometric, body composition and ultrasonographic measurements were compared using independent samples *t*-test for parametric variables, or Mann–Whitney U-test for non-parametric variables. Proportions were compared using a Chi-square test. Correlation analyses were performed using Pearson or Spearman correlation coefficients. Linear regression analysis was performed to evaluate the associations of anthropometrics and body composition variables with intramuscular adipose tissue and echo intensity. To adjust the analyses and minimize bias, the covariates age and sex were added to the model. Multicollinearity was assessed using the Variance Inflation Factor (VIF), and model assumptions were verified through visual inspection of residual plots and normality tests of the residuals. All statistical analysis was performed using IBM SPSS Statistics for Macintosh (version 31.0.0.0, Armonk, NY, USA: IBM Corp), and the level of significance was set at α ≤ 0.05. Although some ultrasonographic variables, particularly echo intensity (EI), exhibited mild deviation from normality, we ensured that the assumptions required for linear regression were adequately met. Specifically, we assessed model residuals through visual inspection of residual plots and conducted normality tests of the residuals. These diagnostics confirmed the approximate normal distribution and homoscedasticity of residuals across models. Therefore, we proceeded with linear regression appropriately.

## 3. Results

The study included 294 participants (153 boys and 141 girls). Mean age was 11.2 ± 2.69 years without differences by sex (*p* = 0.351). In total, 83.7% of the participants exhibited obesity and 16.3% were overweight (Table 1). There were differences in anthropometric variables by sex group, with higher height and weight in the boys’ group. Obesity was more frequent among boys (83.7%), while girls had a higher prevalence of overweight (25.9%; *p* = 0.012). Among circumferential measurements, boys presented higher values in wrist, neck, tight mid-upper arm, mid-calf, and waist measurements (all *p* < 0.05), whereas hip circumference did not differ significantly between sexes. Skinfold measurements were largely similar, except for tight mid-upper arm skinfolds, which were higher in boys (*p* = 0.043).

In body composition estimations, boys showed greater soft lean mass, fat-free mass, musculoskeletal mass, bone mineral content, body cell mass, and total body water compared with girls (*p* < 0.05) (Table 1). Body fat percentage, visceral fat area, and phase angle did not differ significantly between sexes.

**Figure 2:**
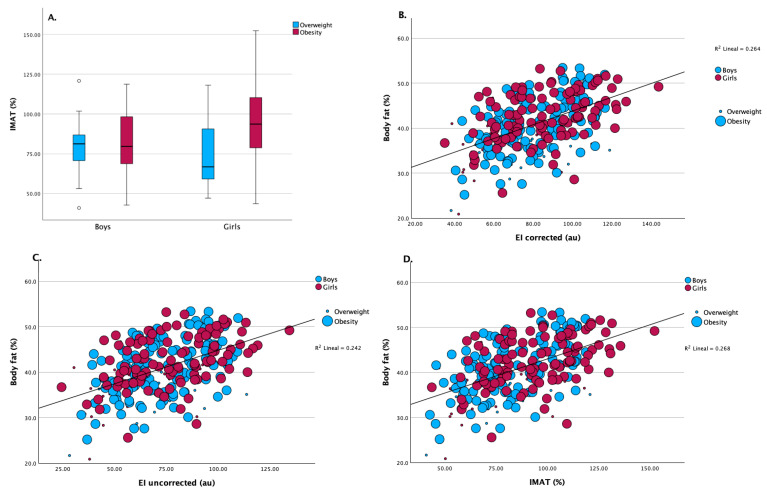
(**A**) Intramuscular adipose tissue (IMAT, %) distribution between boys and girls, stratified by overweight and obesity status. Girls exhibited significantly higher IMAT values than boys in both weight categories (*p* < 0.001 for overweight; *p* = 0.005 for obesity). (**B**) Scatterplot showing the association between corrected echo intensity and body fat percentage. A significant positive correlation was observed (R^2^ = 0.264, *p* < 0.001), consistent across sexes and weight groups. (**C**) Relationship between uncorrected EI values and body fat percentage. The correlation remained statistically significant, though slightly weaker (R^2^ = 0.242, *p* < 0.001). (**D**) Association between IMAT (%) and body fat percentage. A significant positive correlation was observed (R^2^ = 0.268, *p* < 0.001), indicating that higher IMAT is associated with increased adiposity.

Muscle ultrasound assessments indicated comparable quadriceps muscle thickness and subcutaneous adipose tissue thickness between boys and girls. However, girls exhibited significantly higher values of intramuscular adipose tissue (IMAT) percentage compared to boys (91.7 [108.6] % vs. 80.3 [79.9] %; *p* = 0.007) as shown in Table 1 and Figure 2A.

Correlation analyses (Table 2) identified significant associations between body composition parameters and muscle ultrasound outcomes. In both sexes, body fat percentage correlated positively with EI corrected (boys: ρ = 0.49, *p* < 0.01; girls: ρ = 0.52, *p* < 0.01), EI uncorrected (boys: ρ = 0.47, *p* < 0.01; girls: ρ = 0.51, *p* < 0.01), and IMAT (boys: ρ = 0.50, *p* < 0.01; girls: ρ = 0.52, *p* < 0.01). In girls, BMI percentile demonstrated positive correlations with EI corrected (ρ = 0.38, *p* < 0.01), EI uncorrected (ρ = 0.36, *p* < 0.01), and IMAT (ρ = 0.38, *p* < 0.01). Associations between circumferential measurements and muscle ultrasound parameters were limited, although waist circumference correlated positively with EI corrected and IMAT in girls. Both tricipital and subscapular skinfolds were positively correlated with EI measures and IMAT across sexes. Conversely, soft lean mass, fat-free mass, and musculoskeletal mass were inversely correlated with EI and IMAT in both groups.

A positive relationship between body fat percentage and EI was found in the corrected (R^2^ = 0.264) and uncorrected (R^2^ = 0.242) linear analysis, as shown in Figure 2A and Figure 2B, respectively, indicating that increased EI is associated with higher body fat, regardless of sex. Figure 2D shows a similar positive association between body fat percentage and IMAT (R^2^ = 0.268). Girls contributed more data points at higher IMAT and body fat levels, consistent with the overall higher IMAT observed in this group. These findings reinforce the link between higher adiposity and elevated muscle ultrasound fat infiltration parameters.

Linear regression analyses (Table 3) identified body fat percentage as the principal predictor of EI corrected in boys (β = 0.57, 95% CI: 0.67 to 1.60, *p* < 0.001), explaining 31% of the variance (R^2^ = 0.31). Among girls, BMI percentile (β = 0.56, 95% CI: 1.34 to 3.95, *p* < 0.001), subcutaneous adipose tissue thickness (β = 0.26, 95% CI: 0.09 to 1.02, *p* = 0.021), neck circumference (β = –0.67, 95% CI: −4.04 to −1.11, *p* < 0.001), and relaxed mid-upper arm circumference (β = 0.55, 95% CI: 0.40 to 3.10, *p* = 0.012) collectively explained 59% of the variance in EI corrected (R^2^ = 0.59). High intercept values reflect the centering of variables and model structure, especially when categorical or interaction terms are involved. Collinearity statistics showed that all predictors had VIF values below 5 (range: 1.000–4.505), indicating that multicollinearity was not a significant concern in the regression models. Additional VIF tables, residual plots, and multicollinearity diagnostics are available upon request to support transparency and reproducibility.

Comparable predictors emerged for EI uncorrected and IMAT. In boys, body fat percentage remained the primary determinant for EI uncorrected (β = 0.70, 95% CI: 0.94 to 1.77, *p* < 0.001) and IMAT (β = 0.57, 95% CI: 0.66 to 1.60, *p* < 0.001). In girls, BMI percentile and subcutaneous adipose thickness continued to be significant predictors of both EI uncorrected and IMAT, with R^2^ values ranging from 0.51 to 0.59.

Analyses stratified by age group (Supplementary Table S1) revealed significant differences in anthropometric, body composition, and muscle ultrasound parameters between children and adolescents in both sexes. Adolescents presented higher values in height, weight, BMI, circumferential measures, indices of muscle mass, and strength assessments compared to children (*p* < 0.001 for all comparisons). Among girls, IMAT percentage and echo intensity measures were significantly lower in adolescents compared to children (IMAT: 82.1 [108.6] % vs. 105.1 [85.4] %, *p* < 0.001). No significant differences in IMAT were observed between age groups in boys.

## 4. Discussion

This study evaluates the association between different anthropometric measurements, muscle strength, and body composition estimations with ultrasound-derived indicators such as muscle thickness, proportion of IMAT, and EI in a sample of Mexican children and adolescents with overweight or obesity. The reviewed literature confirms a consistent relationship between obesity-related alterations in body composition and muscle ultrasound parameters in children and adolescents. Muscle thickness (MT), a surrogate of muscle mass, has shown moderate-to-strong correlations with functional measures such as strength and physical performance across a variety of pediatric cohorts [1,2,8,10,25,28]. Studies have demonstrated that increased adiposity—particularly VAT and SAT—is associated with changes in MT and, more prominently, with elevated EI, indicative of compromised muscle quality [11,13,19,27,46].

One of the central insights from Akima et al. [46] is the role of VAT and age in predicting increased intramuscular adiposity, reflected by higher EI in abdominal and thigh musculature. This aligns with findings from Abe et al. [1], which highlight MT as a valid indicator of muscle cross-sectional area and strength capacity. These associations are echoed in both pediatric and adult populations but hold critical relevance in children due to the early onset of musculoskeletal decline linked to obesity and sedentary lifestyles [14,20,26]. Echo intensity, while promising as a biomarker for muscle quality, presents interpretation challenges. EI values are influenced by several confounders including SAT thickness, probe pressure, gain settings, tissue hydration, and operator technique [15,18,38,39]. Acuña-Pardo et al. and other groups [5,7,16] noted the moderate predictive power of EI for muscle weakness and poor functional status in pediatric cohorts, emphasizing the need for combined assessments using MT, physical performance tests, and metabolic parameters. The reliability of ultrasound measurements across studies varied. While MT generally exhibited high intra- and inter-rater reliability [9,10,22], EI showed more variability, underscoring the importance of standardized imaging protocols and calibration [12,37,38]. Advances in ultrasound software and artificial intelligence may further improve measurement accuracy and reduce operator dependence.

Clinically, elevated EI in children with obesity has been linked to increased markers of systemic inflammation, insulin resistance, and hepatic steatosis, making it a useful surrogate for broader metabolic risk [30,31,32,33,36]. This is particularly critical in children exhibiting poor dietary habits, low physical activity, or established comorbidities. Integrating EI with body composition profiling may help identify children at risk for sarcopenic obesity, a condition defined by high fat mass and poor muscle quality [13,24,42]. Several intervention studies support the responsiveness of EI to treatment. Reductions in EI following structured exercise or nutritional interventions have been associated with improvements in muscle strength and metabolic outcomes [34,35,36,41]. However, most of these findings are from small-scale or short-term studies, and larger longitudinal studies are needed to validate the prognostic utility of EI and MT. Despite these promising findings, several knowledge gaps persist. Normative values for EI in pediatric populations are limited, particularly across diverse ethnicities, pubertal stages, and disease phenotypes [21,30,47]. Few studies have systematically tracked ultrasound muscle parameters across time in relation to growth trajectories or chronic disease development. Moreover, while MT reflects structural adaptations, its functional implications are context-dependent and should not be interpreted in isolation.

Ultrasound-derived measurements of muscle thickness and echo intensity provide valuable, non-invasive insights into the musculoskeletal and metabolic health of children and adolescents with obesity. Muscle thickness effectively represents muscle size, while echo intensity serves as an indicator of muscle quality and functional potential, though interpretation requires caution due to influencing factors. Combined, these modalities hold promise for enhancing early detection, risk stratification, and personalized interventions in pediatric obesity. To fully leverage their utility, efforts toward standardization, the expansion of normative reference data, and integration into clinical practice pathways remain crucial. Future research should focus on longitudinal analyses and the integration of functional outcomes to establish the prognostic value of these ultrasound parameters and their role in guiding personalized interventions.

This study has several limitations that should be considered when interpreting the findings. First, due to its cross-sectional design, causality cannot be established between adiposity and muscle quality outcomes. Second, pubertal staging was not assessed, which may have introduced variability in body composition and muscular development across the wide age range of participants. Although BMI percentiles are inherently adjusted for age and sex, we observed statistically significant differences between boys and girls within our sample. These discrepancies may reflect sex-specific variations in fat distribution, hormonal influences, or pubertal progression. Girls exhibited higher fat mass and intramuscular adipose tissue (IMAT), while boys had greater lean and musculoskeletal mass. Such differences likely influenced the observed associations between BMI percentile and ultrasound-derived muscle quality indicators, such as echo intensity (EI) and IMAT. EI may be influenced by technical factors such as probe compression, gain settings, and biological variability including subcutaneous adipose tissue (SAT), hydration status, and pubertal stage. These factors limit the generalizability and comparability of EI values across studies and populations. Accordingly, sex-related patterns in body composition should be considered when interpreting BMI percentile outcomes in pediatric populations. Additionally, the absence of functional performance testing limits the ability to relate the imaging findings directly to muscle function or physical capacity. Finally, the study did not include validation against gold-standard imaging techniques, such as MRI or CT, which—while more precise—are often impractical in large pediatric cohorts due to cost, accessibility, and radiation exposure considerations.

## 5. Conclusions

This study demonstrates significant associations between body composition measurements and muscle ultrasound-derived indicators, particularly echo intensity and intramuscular adipose tissue, in children and adolescents with overweight and obesity. Higher adiposity metrics were linked to poorer muscle quality indicators, suggesting early alterations in muscle architecture in pediatric obesity. Muscle thickness, while reflecting size, did not fully capture muscle quality, highlighting the importance of integrating both morphological and qualitative ultrasound parameters for comprehensive assessment. Although this cross-sectional study supports the association between body composition and muscle quality, the absence of functional performance data and longitudinal follow-up limits conclusions regarding prognostic utility. Nonetheless, muscle ultrasound may offer valuable complementary insights in early clinical assessments of pediatric populations with obesity. These findings underscore the potential utility of ultrasound as a non-invasive tool for early detection of musculoskeletal alterations in pediatric obesity.

## Figures and Tables

**Table 1:** Descriptive characteristics of the full sample of both boys and girls in the study.

	Full Sample (n = 294)	Boys (n = 153)	Girls (n = 141)	*p* Value
Anthropometric parameters				
Age (years) *	11.2 ± 2.6	11.3 ± 2.6	11.2 ± 2.8	0.351 ^a^
Height (cm) *	147.7 ± 14.1	150.0 ± 14.4	145.4 ± 13.3	0.002 ^a^
Weight (kg) *	60.8 ± 19.3	63.4 ± 20.0	58.0 ± 18.2	0.008 ^a^
Body mass index (kg/m^2^) *	27.0 ± 4.6	27.4 ± 4.7	26.7 ± 4.5	0.095 ^a^
Body mass index (percentile) *	96.9 ± 2.7	97.5 ± 2.3	96.4 ± 3.0	<0.001 ^a^
Overweight (%) ^†^	16.3	5.8	10.5	0.012 ^c^
Obesity (%) ^†^	83.7	46.3	37.4	
Height for age				
Normal (%) ^†^	83.7	43.2	40.5	0.016 ^c^
Tall (%) ^†^	8.8	6.5	2.4	
Marginally stunted (%) ^†^	7.5	2.4	5.1	
Circumferences (cm)				
Wrist *	15.5 ± 1.4	15.9 ± 1.4	15.2 ± 1.3	<0.001 ^a^
Neck *	35.0 ± 19.1	37.1 ± 26.1	32.8 ± 3.0	0.026 ^a^
Relaxed mid-upper arm *	30.7 ± 4.5	31.0 ± 4.5	30.4 ± 4.5	0.108 ^a^
Tight mid-upper arm *	30.6 ± 4.5	31.0 ± 4.6	30.1 ± 4.4	0.043 ^a^
Mid-thigh *	56.0 ± 8.1	56.3 ± 7.8	55.8 ± 8.5	0.286 ^a^
Mid-calf *	34.8 ± 4.6	35.4 ± 4.4	34.2 ± 4.7	0.017 ^a^
Waist *	86.2 ± 11.7	89.5 ± 11.8	82.8 ± 10.5	<0.001 ^a^
Hip *	95.1 ± 13.8	95.6 ± 14.2	94.6 ± 13.3	0.277 ^a^
Skinfold measurements				
Tricipital *	23.4 ± 6.5	23.3 ± 6.3	23.4 ± 6.8	0.449 ^a^
Subscapular *	26.3 ± 9.3	25.8 ± 8.8	26.8 ± 9.8	0.183 ^a^
Sum of skinfolds *	49.7 ± 14.5	49.2 ± 13.3	50.3 ± 15.8	0.262 ^a^
Strength measurements				
Right hand grip **^§^**	11.0 (41.5)	12.0 (38.0)	11.0 (41.5)	0.187 ^b^
Left hand grip **^§^**	10.0 (35.5)	10.0 (35.0)	9.0 (31.5)	0.304 ^b^
Back and leg **^§^**	36.0 (96.0)	38.0 (96.0)	35.0 (61.0)	0.257 ^b^
BIA parameters				
Body fat (%) **^§^**	41.1 (34.6)	40.9 (31.7)	41.2 (34.6)	0.218 ^b^
Body fat (kg) **^§^**	23.7 (57.3)	23.9 (50.1)	23.6 (57.3)	0.200 ^b^
Visceral fat area (cm^2^) **^§^**	119.1 (237.9)	120.3 (220.1)	118.8 (237.9)	0.411 ^b^
Soft lean mass (kg) **^§^**	32.3 (49.6)	32.7 (49.2)	31.2 (41.7)	0.016 ^b^
Fat-free mass (kg) **^§^**	34.2 (53.0)	34.7 (52.5)	33.0 (44.4)	0.015 ^b^
Musculoskeletal mass (kg) **^§^**	18.3 (32.2)	18.5 (32.0)	17.7 (28.3)	0.025 ^b^
Bone mineral content (kg) **^§^**	1.9 (3.6)	2.0 (3.6)	1.9 (2.9)	0.020 ^b^
Body cell mass (kg) **^§^**	22.3 (35.3)	22.6 (35.2)	21.7 (31.0)	0.025 ^b^
Total body water (L) **^§^**	25.2 (38.3)	25.5 (37.9)	24.3(31.8)	0.013 ^b^
Extracellular water (L) **^§^**	9.6 (14.1)	9.8 (13.8)	9.2 (11.7)	0.008 ^b^
Intracellular water (L) **^§^**	15.6 (24.6)	15.8 (24.5)	15.1 (21.6)	0.019 ^b^
Body phase angle (°) **^§^**	5.4 (4.4)	5.4 (3.6)	5.5 (4.4)	0.110 ^b^
Muscle ultrasound parameters				
Quadriceps muscle thickness (mm) **^§^**	40.5 (37.2)	40.6 (35.0)	40.5 (37.2)	0.643 ^b^
Subcutaneous adipose thickness (mm) **^§^**	18.8 (51.3)	18.5 (38.9)	19.1 (49.0)	0.511 ^b^
IMAT (%) **^§^**	82.8 (111.3)	80.3 (79.9)	91.7 (108.6)	0.007 ^b^
EI uncorrected (au) **^§^**	72.5 (110.1)	71.4 (85.6)	75.2 (110.1)	0.679 ^b^
EI corrected (au) **^§^**	78.9 (108.7)	78.0 (80.6)	83.0 (108.7)	0.679 ^b^

The data are presented as * mean ± standard deviation, ^§^ median (interquartile range, IQR) or ^†^ proportions. The statistical analyses utilized were ^a^ independent samples *t*-test; ^b^ Mann–Whitney U-test; ^c^ Chi-square test. BIA: bioelectrical impedance analysis; IMAT: proportion of intramuscular adipose tissue; EI: echo intensity expressed in arbitrary units.

**Table 2:** Correlations between anthropometry measurements, body composition estimations, and ultrasonographic parameters by sex group.

Variables	Echo Intensity Corrected (au)		Echo Intensity Uncorrected (au)		IMAT (%)	
	Boys	Girls	Boys	Girls	Boys	Girls
Age (years)	−0.22 **	−0.21 *	−0.21 **	−0.23 **	−0.22 **	−0.21 *
Height (cm)	−0.21 *	−0.16	−0.21 *	−0.17 *	−0.21 *	−0.16
Weight (kg)	−0.09	−0.03	−0.10	−0.06	−0.09	−0.02
Body mass index (kg/m^2^)	0.13	0.12	0.10	0.09	0.13	0.13
Body mass index (percentile)	0.16	−038 **	0.15	0.36 **	0.16 *	0.38 **
Circumferences (cm)						
Wrist	−0.06	0.00	−0.07	−0.02	−0.06	0.00
Neck	0.11	0.01	0.11	−0.02	0.10	0.01
Relaxed mid-upper arm	0.02	−0.00	0.01	−0.03	0.02	−0.00
Tight mid-upper arm	0.13	0.12	0.11	0.09	0.13	0.12
Mid-thigh	0.02	−0.03	0.00	−0.06	0.02	−0.02
Mid-calf	−0.02	−0.03	−0.03	−0.06	−0.02	−0.03
Waist	0.14	0.21 *	0.13	0.18 *	0.14	0.21 *
Hip	−0.01	0.05	−0.02	0.02	−0.01	0.05
Skinfold measurements						
Tricipital	0.28 **	0.24 **	0.27 **	0.23 **	0.28 **	0.24 **
Subscapular	0.19 *	0.35 **	0.18 *	0.33 **	0.20 *	0.35 **
Sum of skinfolds	0.26 **	0.32 **	0.25 **	0.30 **	0.26 **	0.32 **
Strength measurements ^§^						
Right hand grip	0.03	0.05	0.04	0.05	0.02	0.30 **
Left hand grip	0.07	0.10	0.09	0.09	0.06	0.39 **
Back and leg grip	−0.00	0.10	0.02	0.13	−0.01	0.39 **
BIA parameters ^§^						
Body fat (%)	0.49 **	0.52 **	0.47 **	0.51 **	0.50 **	0.52 **
Body fat (kg)	0.16 *	0.12	0.16	0.10	0.17 *	0.12
Visceral fat area (cm^2^)	0.31 **	0.31 **	0.30 **	0.29 **	0.31 **	0.31 **
Soft lean mass (kg)	−0.17 *	−0.23 **	−0.17 *	−0.25 **	−0.18 *	−0.23 **
Fat-free mass (kg)	−0.17 *	−0.23 **	−0.17 *	−0.25 **	−0.17 *	−0.23 **
Musculoskeletal mass (kg)	−0.17 *	−0.24 **	−0.16	−0.25 **	−0.17 *	−0.23 **
Bone mineral content (kg)	−0.22 **	−0.28 **	−0.22 **	−0.29 **	−0.23 **	0.27 **
Body cell mass (kg)	−0.17 *	−0.24 **	−0.16	−0.26 **	−0.17 *	−0.23 **
Total body water (L)	−0.17 *	−0.23 **	−0.16	−0.25 **	−0.17 *	0.223 **
Extracellular water (L)	−0.17 *	−0.22 *	−0.16	−0.24 **	−0.17 *	−0.21 *
Intracellular water (L)	−0.16 *	−0.23 **	−0.16	−0.25 **	−0.17 *	−0.23 **
Body phase angle (°)	−0.22 **	−0.26 **	−0.21 **	−0.26 **	−0.23 **	−0.26 **
Muscle ultrasound parameters ^§^						
Quadriceps muscle thickness (mm)	−0.17 *	−0.08	−0.16	−0.09	−0.17 *	−0.08
Subcutaneous adipose thickness (mm)	−0.16	−0.04	−0.10	−0.08	0.17 *	−0.04

*p* value: * *p* = 0.05; ** *p* = 0.01. ^§^ Spearman correlations. au: arbitrary units; IMAT: Intramuscular adipose tissue.

**Table 3:** Linear regression models predicting EI_corrected_, EI_uncorrected_, and IMAT.

	Boys		Girls		
	β (95% CI)	*p* Value	Variable	β (95% CI)	*p* Value
EI_corrected_					
Intercept	48.7 (17.4, 80.0)	0.003	Intercept	−189.3 (−315.1, −63.5)	0.004
Body fat (%)	0.57 (0.67, 1.6)	<0.001	BMI (percentile)	0.56 (1.3, 3.9)	<0.001
Subcutaneous adipose thickness (mm)	0.26 (0.09, 1.02)	0.021		R = 0.56, R^2^ = 0.31	
Neck circumference	−0.67 (−4.04, −1.1)	<0.001			
Relaxed mid-upper arm circumference	0.55 (0.40, 3.10)	0.012			
	R = 0.77, R^2^ = 0.59				
EI_uncorrected_					
Intercept	−2.45 (−22.5, 17.5)	0.806	Intercept	−2.7 (−33.2, 27.6)	0.855
Body fat (%)	0.7 (0.9, 1.7)	<0.001	Body fat (%)	0.55 (0.73, 2.2)	<0.001
Subcutaneous adipose thickness (mm)	0.25 (0.08, 0.97)	0.021		R = 0.55, R^2^ = 0.31	
	R = 0.71, R^2^ = 0.51				
IMAT					
Intercept	52.16 (20.48, 83.84)	0.002	Intercept	−181.7 (−307.3, −56.1)	0.006
Body fat (%)	0.57 (0.66, 1.60)	<0.001	BMI (percentile)	0.56 (1.35, 3.96)	<0.001
Subcutaneous adipose thickness (mm)	0.26 (0.09, 1.03)	0.020		R = 0.56, R^2^ = 0.31	
Neck circumference	−0.67 (−4.10, −1.12)	<0.001			
Relaxed mid-upper arm circumference	0.55 (0.39, 3.12)	0.013			
	R = 0.76, R^2^ = 0.59				

Values are expressed as standardized coefficients (β). BMI: Body mass index; IMAT: Intramuscular adipose tissue.

## Data Availability

The original contributions presented in this study are included in the article. Further information can be available after inquiries to the corresponding authors.

## Supplementary Materials

The following supporting information can be downloaded at: https://www.mdpi.com/article/10.3390/nu17162659/s1, Table S1: Descriptive statistics of children and adolescents analyzed by sex.

## Abbreviations

The following abbreviations are used in this manuscript:

IMAT	Intramuscular Adipose Tissue
au	Arbitrary Units
BIA	Bioelectrical Impedance Analysis
BMI	Body Mass Index
CDC	Centers for Disease Control and Prevention
cm	Centimeter(s)
cm^2^	Square Centimeter
Co.	Company
Corp	Corporation
dB	Decibel(s)
DXA	Dual-energy X-ray Absorptiometry
EI	Echo intensity
GE	General Electric
IBM	International Business Machines
IC	Confidence Interval.
ISAK	International Society for the Advancement of Kinanthropometry
Kg	Kilogram(s)
kg/m^2^	Kilograms per Square Meter
L	Liter(s)
Ltd.	Limited
m^2^	Square Meter(s)
MHz	Megahertz
mm	Millimeter(s)
MT	Muscle Thickness
NAFLD	Non-Alcoholic Fatty Liver Disease
NIH	National Institutes of Health
NY	New York
ROI	Region of Interest
SAT	Subcutaneous Adipose Tissue
SD	Standard Deviation
SPSS	Statistical Package for the Social Sciences
USA	United States of America
VAT	Visceral Adipose Tissue
