# Supplementary material for: Associations Between Body Composition Measurements and Muscle Ultrasound Parameters Amongst Children and Adolescents with Overweight and Obesity
Source: Nutrients. 2025 Aug 17;17(16):2659. doi: 10.3390/nu17162659 (PMC12389388; doi:10.3390/nu17162659)
Supplement: Supplementary file 1 [file nutrients-17-02659-s001.zip › nutrients-3783332-supplementary.pdf]

Table Supplementary S1. Descriptive statistics of children and adolescents analyzed by sex.

	Boys			Girls		
	Children (n = 47)	Adolescents (n = 106)	*P* value	Children (n = 56)	Adolescents (n = 85)	*P* value
**Anthropometric parameters**						
Age (years)	8.4 ± 1.1	12.5 ± 1.9	<0.001*	8.40 ± 1.1	13.0 ± 2.0	<0.001*
Height (cm)	135.3 ± 10.1	156.5 ± 10.7	<0.001*	132.6 ± 8.5	153.8 ± 8.3	<0.001*
Weight (kg)	44.8 ± 11.4	71.7 ± 17.3	<0.001*	42.6 ± 9.8	68.2 ± 14.9	<0.001*
Body mass index (kg/m^2)	24.1 ± 3.7	28.9 ± 4.3	<0.001*	23.9 ± 3.3	28.6 ± 4.2	<0.001*
Body mass index (percentile)	97.6 ± 2.2	97.4 ± 2.4	0.260	96.7 ± 3.4	96.1 ± 2.8	0.226
Overweight (%)[†]	3.3	7.8	0.901 [b]	6.4	15.6	0.169 [b]
Obesity (%)[†]	27.5	61.4		33.3	44.7	
Height for age						
Normal (%)[†]	24.8	58.2		34.0	50.4	
Tall (%)[†]	5.2	7.2	0.353 [b]	3.5	1.4	0.067 [b]
Marginally stunted (%)[†]	0.7	3.9		2.1	8.5	
**Circumferences (cm)**						
Wrist	15.0 ± 1.2	16.4 ± 1.2	<0.001*	14.4 ± 1.2	15.6 ± 1.2	<0.001*
Neck	32.2 ± 2.8	39.3 ± 31.1	0.060	30.7 ± 2.4	34.1 ± 2.7	<0.001*
Relaxed mid-upper arm	26.9 ± 3.1	32.9 ± 3.8	<0.001*	27.1 ± 3.3	32.6 ± 3.7	<0.001*
Tight mid-upper arm	27.2 ± 3.2	32.7 ± 4.0	<0.001*	27.3 ± 3.6	32.0 ± 3.9	<0.001*
Mid-thigh	49.2 ± 5.1	59.5 ± 6.6	<0.001*	49.2 ± 6.1	60.1 ± 7.0	<0.001*
Mid-calf	31.5 ± 3.3	37.1 ± 3.7	<0.001*	30.4 ± 3.2	36.8 ± 3.8	<0.001*
Waist	80.0 ± 9.8	93.7 ± 10.1	<0.001*	76.7 ± 9.3	86.8 ± 9.3	<0.001*
Hip	83.2 ± 12.5	101.1 ± 11.1	<0.001*	83.6 ± 8.7	101.9 ± 10.6	<0.001*
**Skinfold measurements**						
Tricipital	22.0 ± 6.4	23.9 ± 6.2	0.037*	21.7 ± 5.8	24.6 ± 7.2	0.007*
Subscapular	23.0 ± 9.2	27.1 ± 8.3	0.004*	24.0 ± 8.3	28.7 ± 10.3	0.003*
Sum of skinfolds	45.0 ± 13.3	51.1 ± 12.9	0.004*	45.7 ± 13.3	53.3 ± 16.6	0.002*
**Strength measurements[§]**						
Right hand grip	6.0 (19.0)	14.5 (38.0)	<0.001 [a]*	6.0 (14.5)	13.0 (39.0)	<0.001 [a]*
Left hand grip	5.0 (17.0)	13.0 (34.0)	<0.001 [a]*	5.0 (13.5)	12.0 (29.0)	<0.001 [a]*
Back and leg	31.0 (39.0)	45.0 (96.0)	0.005 [a]*	28.0 (38.0)	41.0 (61.0)	<0.001 [a]*
**BIA parameters[§]**						
Body fat (%)	40.5 (29.7)	40.9 (31.7)	0.309 [a]	42.0 (32.3)	40.8 (29.9)	0.918

Body fat (kg)	18.0 (27.2)	27.2 (42.3)	<0.001 [a]*	17.3 (28.4)	26.5 (48.3)	<0.001 [a]*
Visceral fat area (cm^2)	99.0 (162.3)	131.3 (198.2)	<0.001 [a]*	97.4 (175.5)	126.4 (218.1)	<0.001 [a]*
Soft lean mass (kg)	25.5 (25.5)	36.8 (41.8)	<0.001 [a]*	22.8 (19.2)	36.2 (33.0)	<0.001 [a]*
Fat-free mass (kg)	27.0 (26.9)	38.9 (44.7)	<0.001 [a]*	24.2 (20.4)	38.4 (35.2)	<0.001 [a]*
Musculoskeletal mass (kg)	13.9 (16.3)	21.1 (27.2)	<0.001 [a]*	12.4 (12.3)	21.1 (22.8)	<0.001 [a]*
Bone mineral content (kg)	1.5 (1.5)	2.3 (3.0)	<0.001 [a]*	1.3 (1.2)	2.3 (2.3)	<0.001 [a]*
Body cell mass (kg)	17.5 (17.9)	25.4 (29.9)	<0.001 [a]*	15.8 (13.4)	25.3 (25.1)	<0.001 [a]*
Total body water (L)	19.8 (19.8)	28.7 (32.2)	<0.001 [a]*	17.8 (15.0)	28.1 (25.0)	<0.001 [a]*
Extracellular water (L)	7.6 (7.4)	11.0 (11.7)	<0.001 [a]*	6.8 (5.6)	10.6 (9.0)	<0.001 [a]*
Intracellular water (L)	12.2 (12.4)	17.7 (20.9)	<0.001 [a]*	11.1 (9.4)	17.6 (17.5)	<0.001 [a]*
Body phase angle (º)	5.1 (2.2)	5.5 (3.1)	<0.001 [a]*	5.2 (2.4)	5.7 (3.9)	<0.001 [a]*
**Muscle ultrasound parameters**§						
Quadriceps muscle thickness (mm)	37.3 (32.9)	41.5 (34.5)	0.008 [a]*	37.9 (24.2)	43.7 (33.0)	<0.001 [a]*
Subcutaneous adipose thickness (mm)	17.2 (22.8)	19.2 (38.0)	0.057 [a]	18.1 (18.2)	19.7 (49.0)	0.127 [a]
IMAT (%)	82.1 (67.5)	79.0 (77.8)	0.695 [a]	105.1 (85.4)	82.1 (108.6)	<0.001 [a]*
EI uncorrected (au)	73.8 (73.9)	69.7 (81.5)	0.910 [a]	89.6 (81.2)	65.7 (110.1)	<0.001 [a]*
EI corrected (au)	79.5 (68.3)	76.8 (78.2)	0.732 [a]	96.8 (85.1)	73.6 (108.7)	<0.001 [a]*

§*Medians (Interquartile range, IQR); † Proportions; BIA: bioelectrical impedance analysis; IMAT: proportion of intramuscular adipose tissue; EI: echo intensity expressed in arbitrary units;* [a] *Mann-Whitney U test;* [b] *Chi-square test*
